# Artificial Intelligence-Based Arterial Input Function for the Quantitative Assessment of Myocardial Blood Flow and Perfusion Reserve in Cardiac Magnetic Resonance: A Validation Study

**DOI:** 10.3390/diagnostics15182341

**Published:** 2025-09-16

**Authors:** Lara R. van der Meulen, Maud van Dinther, Amedeo Chiribiri, Jouke Smink, CRUCIAL Investigators, Walter H. Backes, Jonathan Bennett, Joachim E. Wildberger, Cian M. Scannell, Robert J. Holtackers

**Affiliations:** 1 Department of Radiology and Nuclear Medicine, Maastricht University Medical Centre, 6229 HX Maastricht, The Netherlands; 2 Cardiovascular Research Institute Maastricht (CARIM), Maastricht University, 6200 MD Maastricht, The Netherlands; 3 Department of Neurology, Maastricht University Medical Centre, 6229 HX Maastricht, The Netherlands; 4 School of Biomedical Engineering and Imaging Sciences, King’s College London, St Thomas’ Hospital, London SE1 7EH, UK; 5 Philips Healthcare, 5684 PC Best, The Netherlands; 6 Mental Health & Neuroscience Research Institute, Maastricht University, 6200 MD Maastricht, The Netherlands; 7 Institute of Cardiovascular Science, University College London, London WC1E 6BT, UK; 8 Department of Biomedical Engineering, Eindhoven University of Technology, 5600 MB Eindhoven, The Netherlands

**Keywords:** artificial intelligence, arterial input function, quantitative myocardial perfusion, cardiac magnetic resonance, myocardial blood flow, myocardial perfusion reserve

## Abstract

**Background/Objectives**: To validate an artificial intelligence-based arterial input function (AI-AIF) deep learning model for myocardial blood flow (MBF) quantification during stress perfusion and assess its extension to rest perfusion, enabling myocardial perfusion reserve (MPR) calculation. **Methods**: Sixty patients with or at risk for vascular cognitive impairment, prospectively enrolled in the CRUCIAL consortium, underwent quantitative stress and rest myocardial perfusion imaging using a 3 T MRI system. Perfusion imaging was performed using a dual-sequence (DS) protocol after intravenous administration of 0.05 mmol/kg gadobutrol. Retrospectively, the AI-AIF was estimated from standard perfusion images using a 1-D U-Net model trained to predict an unsaturated AIF from a saturated input. MBF was quantified using Fermi function-constrained deconvolution with motion compensation. MPR was calculated as the stress-to-rest MBF ratio. MBF and MPR estimates from both AIF methods were compared using Bland–Altman analyses. **Results**: Complete stress and rest perfusion datasets were available for 31 patients. A bias of −0.07 mL/g/min was observed between AI-AIF and DS-AIF for stress MBF (median 2.19 vs. 2.30 mL/g/min), with concordant coronary artery disease classification based on the optimal MBF threshold in over 92% of myocardial segments and coronary arteries. Larger biases of 0.12 mL/g/min and −0.30 were observed for rest MBF (1.12 vs. 1.02 mL/g/min) and MPR (2.31 vs. 1.84), respectively, with lower concordance using the optimal MPR threshold (85% of segments, 72% of arteries). **Conclusions**: The AI-AIF model showed comparable performance to DS-AIF for stress MBF quantification but requires further training for accurate rest MBF and MPR assessment.

## 1. Introduction

Myocardial perfusion MRI is a non-invasive imaging technique that allows for the quantification of myocardial blood flow (MBF) [1], typically measured during both stress and rest conditions. Myocardial perfusion reserve (MPR), defined as the ratio of stress to rest MBF, reflects the functional capacity of the heart to increase perfusion during periods of increased demand [2]. Both MBF and MPR are valuable markers for the detection of myocardial ischemia [3]. Their quantification and visualization using color maps is superior to qualitative visual assessment, particularly for less experienced readers [4].

A key challenge in accurately quantifying MBF, however, is the sampling of the arterial input function (AIF), which describes the delivery of contrast agent to the myocardium over time. Difficulties associated with AIF sampling have been well-documented [5,6]. One major difficulty is the non-linear relationship between signal intensity and contrast concentration, which becomes more pronounced at higher contrast concentrations [7,8]. This non-linearity can lead to signal saturation in the AIF and, consequently, to underestimation of the true AIF [1,9].

Two primary methods have been proposed to mitigate signal saturation during quantitative perfusion cardiac MRI: the dual-bolus and dual-sequence (DS) methods. The dual-bolus method involves administering a diluted contrast agent before the regular bolus [10], but the workflow is complex [11] and the AIF and myocardial tissue curves are acquired at different time points [12]. In contrast, the DS method uses a short saturation time to acquire a lower-resolution image slice for AIF measurement, followed by a standard higher-resolution acquisition for imaging the myocardium [10,12]. Although only a single bolus injection is required for this method, the imaging sequence itself is more complex and is currently restricted to specialized research settings. Therefore, there is a demand for an accurate AIF sampling method, while simultaneously imaging the tissue of interest, using a single-bolus, single-sequence acquisition.

Artificial intelligence (AI) has the potential to enhance various aspects of cardiac perfusion MRI. In acquisition, AI can assist with planning imaging planes [13] and accelerating image acquisition [14]. During reconstruction, AI-based image reconstruction can improve image quality from undersampled data [15,16] and enhance low-quality images [17,18,19]. In post-processing, AI—particularly U-Net architectures—has been applied successfully for tasks such as image segmentation [20,21], denoising [22], and motion compensation [23]. Finally, AI can facilitate perfusion quantification by predicting kinetic parameters, such as MBF, from relevant inputs, for example, the AIF [24].

Recently, Scannell et al. (2023) introduced a deep learning model trained to predict the unsaturated AIF from a saturated single-bolus, single-sequence AIF [12]. Stress MBF values quantified using this AI-based AIF (AI-AIF) were comparable to those estimated using DS-derived AIF (DS-AIF), suggesting that AI-based AIF prediction could provide a more accessible and easily integrable alternative to current AIF sampling methods used in clinical practice. Despite this promising proof-of-concept, the method has yet to be widely evaluated across different centers and acquisition settings and has not been applied to quantifying rest perfusion.

The aim of this study is twofold: first, to validate a deep learning model for quantifying MBF from stress perfusion imaging; and second, to extend its application to rest perfusion, thereby enabling calculation of MPR.

## 2. Materials and Methods

### 2.1. Study Population

This study retrospectively analyzed prospectively acquired data from sixty patients enrolled in the CRUCIAL consortium [25] between December 2020 and October 2023, all of whom were scheduled for quantitative perfusion MRI of both the brain and the heart. All patients either had, or were at risk for, vascular cognitive impairment, and underwent cardiac MRI. More detailed inclusion criteria can be found in Table 1. Exclusion criteria included (a) being under 18 years old, (b) having other major neurological or psychiatric conditions affecting the brain, such as multiple sclerosis, Parkinson’s disease, drug abuse, major cortical stroke, major neuro-trauma, or brain tumors, and (c) having contraindications to MRI, such as implanted devices, gadolinium allergy, severe renal impairment (estimated Glomerular Filtration Rate < 30 mL/min/1.73 m^2^), or claustrophobia, and (d) specific exclusion criteria for cardiac stress perfusion MRI, including asthma and/or chronic obstructive pulmonary disease, slow heart rhythm (<50 beats per minute), atrioventricular block II-III, sick sinus syndrome, prolonged QT-interval, hypotension (systolic blood pressure < 90 mm Hg), or decompensation cordis. The CRUCIAL study was approved by the local ethics committee (NL72696.068.20), and all participants provided written informed consent. The study was conducted according to the principles of the Declaration of Helsinki.

### 2.2. Image Acquisition

Quantitative stress and rest perfusion imaging of the heart was performed using a clinical 3 T MRI system (Ingenia CX, Philips Healthcare, Best, the Netherlands). Perfusion data were acquired in free-breathing for three typical short-axis slices covering the left ventricle: basal, midventricular, and apical. For both stress and rest perfusion, a previously described dual-sequence implementation [26] was used where an additional low-resolution image of the basal slice is acquired each heartbeat to minimize signal saturation in the AIF estimation. Typical sequence parameters of the electrocardiogram-triggered saturation-recovery turbo field echo dual-sequence approach included: echo time 1.0 ms, repetition time 2.2 ms, flip angle 15°, acquired resolution 2.6 × 2.6 mm, reconstructed resolution 1.0 × 1.0 mm, slice thickness 10 mm, water-fat shift 0.3 pixels, and sensitivity encoding factor 2.2. Each slice included two to three proton density-weighted images without saturation preparation, used for the surface coil intensity correction. Stress imaging was performed during adenosine-induced hyperemia (140–210 μg/kg/min, depending on the response to stress). For both stress and rest perfusion, an intravenous injection of 0.05 mmol/kg of gadobutrol (Gadovist, Bayer Pharmaceuticals, Berlin, Germany), injected at 4 mL/s, was administered directly prior to imaging, followed by a 25 mL saline flush at the same injection rate.

### 2.3. AI-AIF

**Figure 1:**
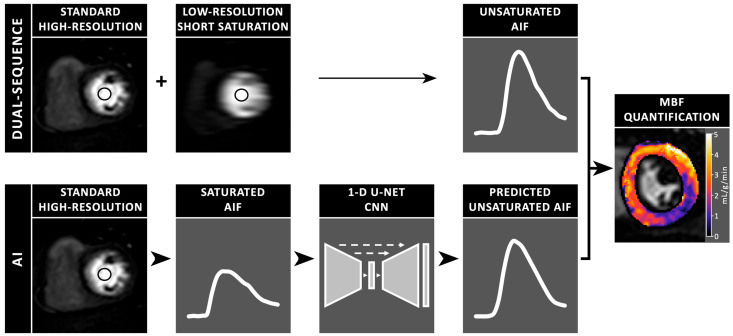
Schematic illustration of myocardial blood flow (MBF) quantification from cardiac perfusion MR images, using two methods to estimate the unsaturated arterial input function (AIF). The dual-sequence method first uses a short saturation time for a lower-resolution image slice to measure the unsaturated AIF, followed by a standard higher-resolution acquisition to image the myocardium. In contrast, the artificial intelligence (AI) model uses the saturated AIF from a standard high-resolution acquisition and predicts the unsaturated AIF through a 1-D U-Net convolutional neural network (CNN).

In addition to estimating the AIF using the additional short saturation time image series, a synthetic AI-AIF was estimated directly from the standard (high-resolution) basal slice using a deep learning model to correct the signal saturation in the AIF sampled from the left ventricular blood pool. The AI-AIF takes the saturated AIF from a standard acquisition as input to a 1-D U-Net model [27] which predicts an unsaturated version (Figure 1). The neural network has been previously trained and validated using a dual-sequence acquisition sequence as ground-truth [12]. Further details on the model architecture and training procedure are described in earlier work by Scannell et al. (2023) [12].

### 2.4. MBF Quantification

Based on the DS-AIF and AI-AIF, a previously described fully automated pixelwise perfusion quantification was performed [28] for each AIF method using Fermi function-constrained deconvolution with motion compensation [29] and AI-based segmentation and image analysis [20]. Subsequently, the right ventricular insertion points were used to summarize pixelwise perfusion values in the 16 myocardial segments [30]. MPR was calculated by dividing the MBF from stress perfusion by the MBF from rest perfusion.

### 2.5. Statistical Analysis

Analyses were performed on both a per-patient basis (where MBF was averaged over all pixels from each patient) and a per-segment basis. Similar analyses were performed for the MPR. Normality of data was assessed visually and using the Shapiro–Wilk [31] or Kolmogorov–Smirnov [32] test for the per-patient and per-segment analyses, respectively. Bland–Altman analysis [33] was performed to evaluate potential biases. The 95% limits of agreement (LoA) and their 95% confidence intervals (CI) [34] were calculated to evaluate the precision.

To assess diagnostic agreement between the AI-AIF and DS-AIF methods, stress MBF and MPR values were classified using optimal thresholds for detecting coronary artery disease (CAD), as defined by Hsu et al. [35]: 1.35 mL/g/min for stress MBF and 1.475 for MPR. Diagnostic classifications were considered concordant if both methods yielded values either above or below the respective threshold for a given myocardial segment or coronary artery. A discordant classification was recorded when one method produced a value above and the other below the threshold. Coronary artery classification was based on the average MBF or MPR of the lowest-perfused myocardial segments within each coronary artery territory [36]. The percentage of agreement between the AI-AIF and DS-AIF methods was reported for both MBF- and MPR-based classifications at the segment and artery levels. All statistical analyses were conducted using SPSS (version 27, IBM, Armonk, NY, USA) and were two-tailed with a significance level of 5%. Data are presented as median and interquartile range (IQR) or percentage, unless otherwise specified.

## 3. Results

### 3.1. Study Cohort

Complete stress and rest perfusion data using the DS method were successfully acquired in 31 of 60 patients (52%). In seven patients, the cardiac MRI scan was aborted prematurely due to claustrophobia and/or panic attacks (*n* = 5) or technical problems (*n* = 2). In 12 patients, no DS method was performed, preventing a direct comparison. Two patients withdrew from the study, and one patient was unable to fit in the MRI scanner with the anterior body coil. Among the 31 included patients, 12 (38.7%) were women, and the median age was 72 years (range 64–79). The baseline characteristics of the study cohort are summarized in Table 2. One myocardial segment from a single patient was excluded due to suboptimal scan planning, as the segment was located at the level of the left ventricular outflow tract, where no myocardium was present.

### 3.2. Stress Myocardial Blood Flow

**Figure 2:**
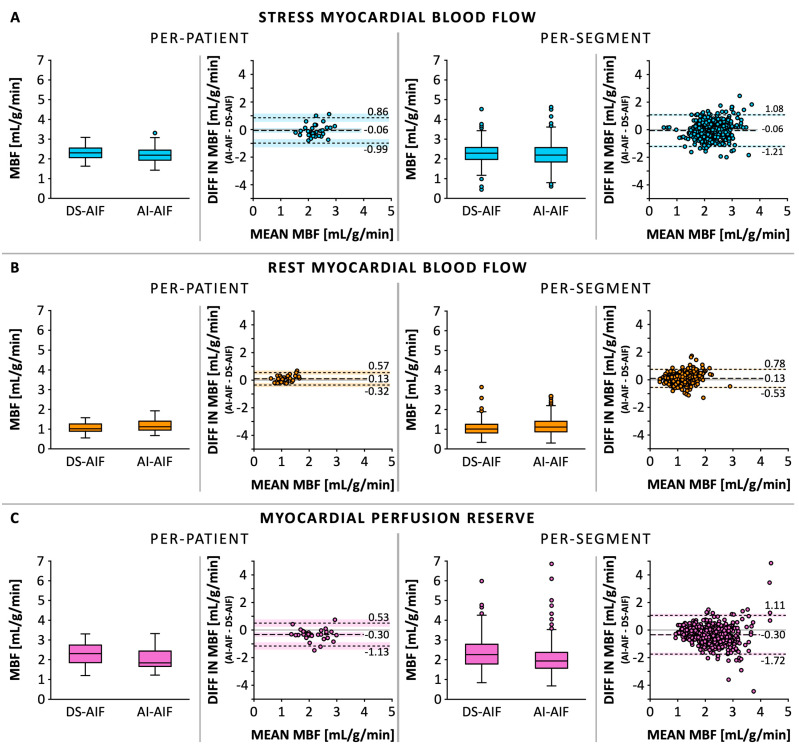
Myocardial blood flow (MBF) quantified using the artificial intelligence-based arterial input function (AI-AIF) and the dual-sequence-derived AIF (DS-AIF) in stress perfusion (**A**) and rest perfusion (**B**). Additionally, the myocardial perfusion reserve (**C**) was calculated by dividing the stress MBF by the rest MBF. The data is displayed on both a per-patient and per-segment level in boxplots, correlation plots, and Bland–Altman plots.

**Figure 3:**
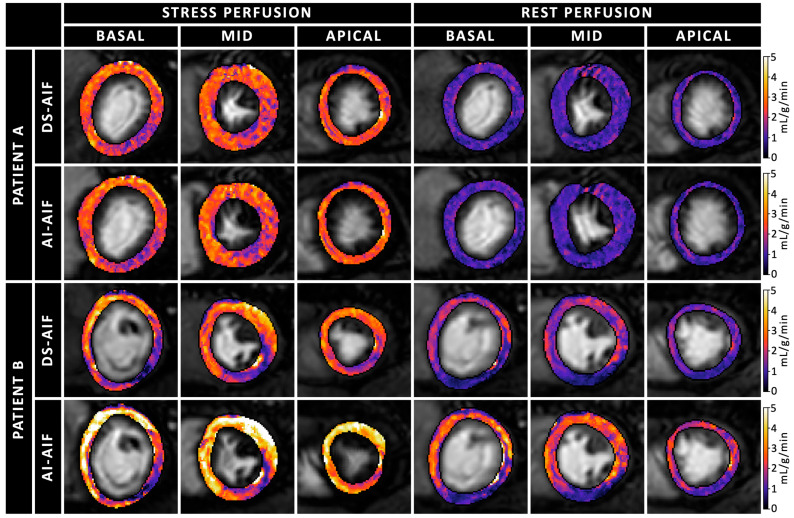
Quantitative pixel-wise myocardial blood flow (MBF) maps during rest and stress conditions in basal, midventricular, and apical short-axis slices, comparing the artificial intelligence-based arterial input function (AI-AIF) and dual-sequence-derived AIF (DS-AIF) in two patients. Patient A (63-year-old man, top panel) shows similar MBF maps for both methods, with only subtle differences. This patient does not have a history of coronary artery disease (CAD). Patient B (60-year-old man, bottom panel) demonstrates more pronounced differences between the two methods. This patient has known CAD, and a clear perfusion defect can be seen in the stress perfusion images.

**Figure 4:**
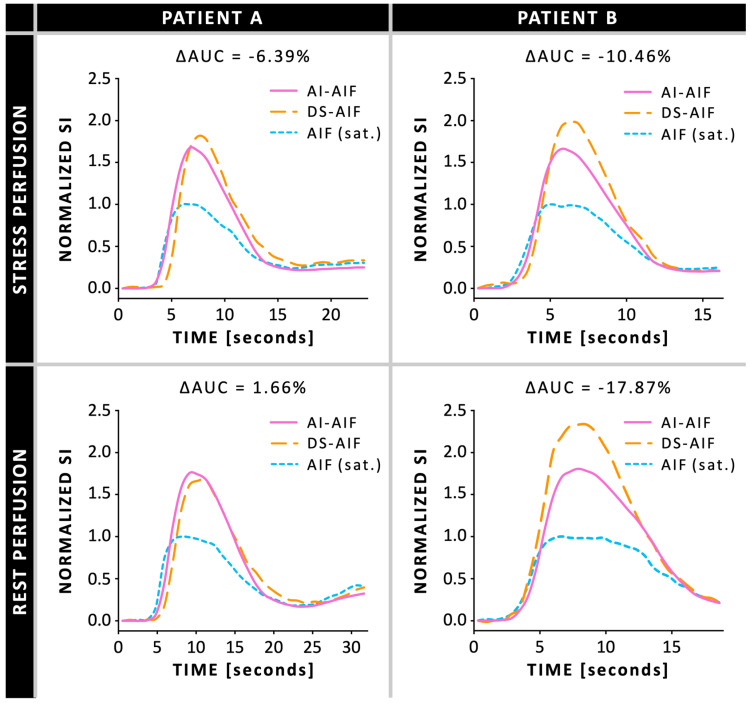
Arterial input function (AIF) curves during ret and stress conditions, comparing the artificial intelligence-based AIF (AI-AIF) and dual-sequence AIF (DS-AIF) to the saturated (sat.) standard AIF, in two patients: patient A is a 63-year-old man and patient B is a 60-year-old man (same as Figure 2). ΔAUC = normalized percentage difference in area under the curve AI-AIF compared to DS-AIF; SI = signal intensity.

The median global stress MBF was 2.20 mL/g/min (IQR 1.93–2.44) when quantified using the AI-AIF, compared to 2.29 mL/g/min (IQR 2.06–2.52) when quantified using the DS-AIF (Figure 2A). Bland–Altman analysis showed a bias of −0.06 mL/g/min in stress MBF between the AI-AIF and DS-AIF approaches (Table 3). On a per-patient level, the 95% CI was −0.24 to 0.11 mL/g/min, with LoAs ranging from −0.99 to 0.86 mL/g/min. On a per-segment level, the 95% CI was −0.12 to −0.01 mL/g/min, with LoAs ranging from −1.21 to 1.08 mL/g/min. In terms of diagnostic accuracy, the classification of CAD based on the optimal stress MBF threshold was concordant between AI-AIF and DS-AIF in 95.2% (471/495) of myocardial segments and 92.5% (86/93) of coronary arteries. Among the 24 discordant segments, 18 (75.0%) were classified as false positives and 6 (25.0%) as false negatives. At the coronary artery territory level, 6 of the 7 (85.7%) discordant coronary arteries were false positives and 1 (14.3%) was a false negative. Examples of pixel-wise MBF maps and AIF curves derived using the AI-AIF and DS-AIF methods are shown in Figure 3; Figure 4, respectively, for two patients.

### 3.3. Rest Myocardial Blood Flow

The median global rest MBF was 1.11 mL/g/min (IQR 0.87–1.41) when quantified using the AI-AIF, compared to 1.01 mL/g/min (IQR 0.81–1.25) when quantified using the DS-AIF (Figure 2B). Bland–Altman analysis showed a bias of 0.13 mL/g/min in rest MBF between the AI-AIF and DS-AIF approaches (Table 3). On a per-patient level, the 95% CI was 0.04 to 0.21 mL/g/min, with LoAs ranging from −0.32 to 0.57 mL/g/min. On a per-segment level, the 95% CI was 0.10 to 0.15 mL/g/min, with LoAs ranging from −0.53 to 0.78 mL/g/min.

### 3.4. Myocardial Perfusion Reserve

The median global MPR was 1.84 (IQR 1.66–2.44) when quantified using the AI-AIF, compared to 2.31 (IQR 1.85–2.74) when quantified using the DS-AIF (Figure 2C). Bland–Altman analysis showed a bias of −0.30 in MPR between the AI-AIF and DS-AIF approaches (Table 3). On a per-patient level, the 95% CI was −0.46 to −0.15, with LoAs ranging from −1.13 to 0.53. On a per-segment level, the 95% CI was −0.37 to −0.24, with LoAs ranging from −1.72 to 1.11. In terms of diagnostic accuracy, the classification of CAD based on the optimal MPR threshold was concordant between AI-AIF and DS-AIF in 85.1% (421/495) of myocardial segments and 72.0% (67/93) of coronary arteries. Among the 74 discordant segments, 55 (74.3%) were classified as false positives and 19 (25.7%) as false negatives. At the artery level, 7 of the 26 (26.9%) discordant coronary arteries were false positives and 19 (73.1%) were false negatives.

## 4. Discussion

This study aimed to validate the AI-AIF model developed by Scannell et al. for quantifying MBF from stress perfusion imaging and to extend its application to also include rest perfusion, allowing the calculation of MPR. The AI-AIF approach performed comparably to the measured DS-AIF for quantifying stress perfusion MBF, showing only minimal bias and minimal impact on the diagnostic classification of CAD. Over 92% of myocardial segments and coronary arteries were concordantly classified across the two approaches. In contrast, larger biases were observed in rest perfusion MBF and MPR calculation, leading to more clinically relevant discordance, with over 14% of segments and coronary arteries misclassified using AI-AIF relative to DS-AIF.

Accurate estimation of AIF has long been a challenge in quantitative (myocardial) perfusion imaging. Ideally, AIF sampling should occur as close to the myocardial region of interest as possible [1,5,37]. Traditionally, however, the AIF was directly measured by sampling blood via an arterial catheter [38], a method that is invasive and limited by poor temporal resolution. Nowadays, the AIF is typically estimated directly from the MRI data and sampled in either the basal left ventricular cavity or aortic root [1]. This approach reduces the risk of partial volume effects and increases the likelihood that the AIF measured in these regions is not a true representation of the AIF in the myocardial region of interest, due to contrast delay and dispersion [5].

Signal saturation of the AIF presents another major challenge. The signal intensity in the blood pool is non-linearly related to contrast concentration. This non-linearity becomes more pronounced as the gadolinium concentration in the blood pool exceeds 1 mmol/L [8]. This signal saturation can lead to systematic underestimation of the AIF, resulting in an overestimation of myocardial perfusion [1]. To ensure accurate measurement of the AIF, it is crucial to correct for or avoid signal saturation effects. Several methods have been proposed to mitigate these effects: using lower doses of contrast agents to reduce the risk of signal saturation, dual-bolus approaches, and dual-sequence methods. Although these methods effectively mitigate signal saturation effects, they come with limitations in terms of contrast-to-noise ratio, complexity, and availability, respectively.

To address these limitations, more advanced methods have been proposed to estimate the unsaturated AIF. One such method, a blind estimation technique proposed by Fluckiger et al. (2009) [6], estimates the AIF indirectly based on tissue concentration data from part of the image combined with an iterative computational approach. The model was later refined by incorporating additional AIF information from the unsaturated portions of the blood pool signal [39]. While the estimated AIF was not significantly different from the dual-sequence AIF, the contrast doses used on these studies (0.01–0.03 mmol/kg) were relatively low compared to those typically used in perfusion MRI (0.05–0.075 mmol/kg), which may limit the generalizability of their findings.

Another method involves retrospectively correcting for the signal saturation effects. For example, Li et al. (2023) [40] proposed a novel post-processing method to retrospectively correct the saturated AIF curve after a single-bolus administration of contrast agent. This approach is simpler than dual-bolus or dual-sequence methods, as it does not require additional bolus injections or special pulse sequences. Additionally, it is independent of the MRI system and pulse sequence settings, provided the imaging signal’s sampling rate is sufficient. When compared to positron emission tomography-based MBF measurements, the corrected AIF resulted in a slight overestimation of MBF (0–6%), whereas the uncorrected AIF caused a large overestimation of MBF (135–312%). Bland–Altman analysis showed a small bias of −0.03 mL/g/min with narrow limits of agreement for the corrected AIF, compared to a larger bias of −3.08 mL/g/min with wide limits of agreement for the uncorrected AIF. These findings suggest that the post-processing method can improve the accuracy of MBF quantification.

The AI-based AIF model by Scannell et al. (2023) [12], as validated in this study, offers another promising solution. Their deep learning model predicts the unsaturated AIF from the saturated AIF obtained from a standard high-resolution image acquisition, eliminating the need for complex acquisition schemes, such as dual-bolus approaches or dual-sequence methods. When comparing the AI-derived AIF with the DS-derived AIF, no significant differences in stress MBF were observed in both the training cohort and an external cohort.

This present validation study supports those findings, showing a slightly smaller bias in quantifying stress MBF between the two methods (−0.06 vs. −0.11), with slightly narrower LoAs. Also, in terms of diagnostic accuracy, the AI-AIF model in the present study showed similar performance, with 92% of CAD classifications based on the stress MBF threshold remaining unchanged compared to DS-AIF, versus 89% reported by Scannell et al. These findings confirm that the AI-AIF model performs well on stress perfusion data. However, the relatively wide 95% CI and the wider-than-expected LoAs compared to literature [41] suggest substantial individual variability in stress MBF measurements, possibly due to the limited sample size, differences between the study population and the population used to train the model, or differences in the acquisition protocol and scanner hardware between the training data and our validation data. Additionally, the AI model systematically overestimated rest MBF and underestimated MPR. This may be due to (i) the AI model being exclusively trained on stress perfusion data, which has higher flow ranges, making it less suitable to handle the lower perfusion levels and smaller signal changes observed at rest, and/or (ii) increased saturation in the rest AIF due to the residual baseline contrast from the previous stress acquisition, which is potentially more challenging to correct for. This also highlights the need for further refinement to enhance the model’s accuracy.

Another important consideration for AIF sampling is reproducibility. Because the AI-AIF model generates the AIF automatically, it eliminates operator-dependent variability and should, in principle, enhance reproducibility compared to manual or semi-automated approaches. However, since no scan–rescan data was included in the present study, the repeatability of MBF estimates using AI-AIF could not be directly assessed. Future studies including test–retest or scan–rescan data are important to confirm the robustness of this AI model in clinical practice.

The complexity and technical challenges of accurately quantifying MBF using perfusion MRI have limited its widespread adoption in clinical practice. The AI-AIF model overcomes these barriers by enabling MBF quantification from a standard single-saturation sequence with a single contrast injection. In addition, the model is open-source, integrates with existing clinical software for quantifying MBF, and may facilitate research into 3D quantitative perfusion by eliminating the need for a DS-AIF, enabling shorter data acquisition windows per cardiac [42]. The quantification process could be further improved by combining the AI-AIF with AI-based perfusion quantification [43]. Another practical advantage is computation efficiency: the quantitative MBF estimates and perfusion maps were calculated and produced in approximately one minute per perfusion series using a standard laptop without a dedicated GPU or optimized implementations. This efficiency suggests that, particularly with dedicated computational resources, the AI-AIF model could be implemented in near real time, further facilitating its integration into routine clinical practice. Finally, the model also enables retrospective analysis of studies performed without dual-bolus or dual-sequence methods, allowing reuse of data from large clinical studies, which were originally based on visual assessment, to further validate quantitative perfusion methods.

### Limitations

This study has certain limitations that need to be discussed. First, the contrast dose used for stress and rest perfusion MRI was lower than the contrast dose used for the stress perfusion imaging that the model was trained on (0.05 mmol/kg vs. 0.075 mmol/kg). This was because patients first underwent a brain MRI with contrast administration, as required by the CRUCIAL study protocol, before proceeding to the cardiac MRI. According to hospital protocol, following the brain MRI, only 0.1 mmol/kg of contrast agent remained available for administration during the cardiac perfusion MRI. The lower administered contrast dose, however, decreased the chance of signal saturation. It did not appear to negatively impact the AI-AIF model’s prediction of the unsaturated AIF during stress perfusion, although it might have contributed to the wider-than-expected LoAs observed in the Bland–Altman plot. Second, although the AI-AIF model showed consistent performance across different hospital cohorts, the MRI systems used in both studies were from the same manufacturer. Evaluating its performance on data acquired from different vendors would provide further insight into the model’s robustness and generalizability. Third, the sample size is relatively small (*n* = 31), which reduces statistical power. For this reason, formal hypothesis testing was not performed; instead, descriptive analyses were conducted using Bland–Altman plots and reporting 95% confidence intervals rather than *p*-values. While these analyses provide insights into agreement and variability, the small sample size limits the generalizability of the findings. Larger-scale studies are needed to confirm the reliability of the AI-AIF model and to provide more robust statistical evidence.

## 5. Conclusions

This study validated an AI-AIF model for quantifying MBF during stress perfusion imaging and sought to extend its application to rest perfusion and MPR. The AI-AIF model performed comparably to the DS-AIF method for stress MBF, with only a small bias between the two methods. However, larger differences were observed for rest MBF and MPR, indicating that the model, having been trained exclusively on stress perfusion data, requires further refinement to enable accurate quantification of rest MBF and MPR. The AI-AIF model offers a promising approach to simplify MBF quantification via a regular single-bolus, single-sequence acquisition, potentially allowing accurate stress MBF assessment in both clinical and research settings and supporting broader clinical adoption of quantitative perfusion MRI.

## Figures and Tables

**Table 1:** Inclusion criteria for patients with vascular cognitive impairment (VCI) and for patients at risk of VCI.

Patients with VCI
VCI due to cSVD, defined as: • Visiting a memory clinic or an outpatient clinic in Neurology; • Cognitive complaints; • Demonstration of cognitive deficit: MoCA < 26 or impairment in at least 1 cognitive domain in neuropsychological assessment; • Imaging evidence of cerebral small vessel disease: ○ Extensive leukoaraiosis on CT; ○ (Early) confluent WMH on MRI (Fazekas score ≥ 2); ○ Multiple punctate WMH on MRI (Fazekas 1) in combination with lacunar infarcts or microbleeds.
Clinical dementia rating scale ≤ 1
**Patients at risk of VCI**
Symptomatic cSVD, defined as: • A history of a clinical lacunar stroke with a compatible lesion on CT or MRI (inclusion only > 3 months after stroke onset to avoid acute stroke effects) • Additional imaging evidence of cerebral small vessel disease: ○ Extensive leukoaraiosis on CT, or ○ (Early) confluent WMH on MRI (Fazekas score ≥ 2), or ○ Lacunar infarct in combination with multiple punctate WMH on MRI (Fazekas 1) or microbleeds.

cSVD = cerebral small vessel disease; CT = computed tomography; MoCA = Montreal Cognitive Assessment; MRI = magnetic resonance imaging; VCI = vascular cognitive impairment; WMH = white matter hyperintensities.

**Table 2:** Baseline characteristics of the study cohort.

	*n* = 31
Age in years, median (range)	72 (64–79)
Women, *n* (%)	12 (38.7)
Weight in kg, mean (SD)	77.2 (16.3)
Height in cm, median (IQR)	172.0 (165.0–182.0)
BMI in kg/m^2^, mean (SD)	25.8 (3.5)
Body surface area, mean (SD)	1.91 (0.3)
eGFR in mL/min/1.73 m^2^, median (IQR)	70.8 (16.6)
Medical history, *n* (%)	
Transient ischemic attack	13 (41.9)
Stroke	15 (48.4)
Hypertension	26 (83.9)
Hypercholesterolemia	25 (80.6)
Atrial fibrillation	1 (3.2)
Obstructive sleep apnea	5 (16.1)
Obesity	6 (19.4)
Chronic kidney disease	9 (29.0)
Diabetes mellitus type 2	9 (29.0)
Coronary artery disease	4 (12.9)
Acute coronary syndrome	2 (6.5)
Percutaneous coronary intervention	2 (6.5)
Coronary artery bypass graft	1 (3.2)
Angina	2 (6.5)
Cognitive impairment	28 (90.3)

BMI = body mass index; eGFR = estimated glomerular filtration rate; IQR = interquartile range; *n* = number of patients; SD = standard deviation.

**Table 3:** Bland–Altman analysis results, including bias, lower and upper limits of agreement, with their 95% confidence intervals on both a per-patient and per-segment level.

		Bias					Lower LoA					Upper LoA			
		Mean		95% CI			LoA		95% CI			LoA		95% CI	
**Stress MBF** Per-patient Per-segment		−0.06 −0.06		−0.24 −0.12	0.11 −0.01		−0.99 −1.21		−1.29 −1.30	−0.69 −1.12		0.86 1.08		0.56 0.99	1.16 1.17
**Rest MBF** Per-patient Per-segment		0.13 0.13		0.04 0.10	0.21 0.15		−0.32 −0.53		−0.47 −0.58	−0.18 −0.48		0.57 0.78		0.43 0.73	0.72 0.83
**MPR** Per-patient Per-segment		−0.30 −0.30		−0.46 −0.37	−0.15 −0.24		−1.13 −1.72		−1.40 −1.83	−0.87 −1.61		0.53 1.11		0.26 1.00	0.80 1.22

Stress and rest myocardial blood flow (MBF) values are expressed in mL/g/min. CI = confidence interval; LoA = limit of agreement; MBF = myocardial blood flow; MPR = myocardial perfusion reserve;

## Data Availability

The data presented in this study are available on request from the corresponding author due to the data being part of a larger ongoing unpublished study.

## Abbreviations

The following abbreviations are used in this manuscript:

AI	Artificial intelligence
AIF	Arterial input function
AI-AIF	Artificial intelligence-based arterial input function
CAD	Coronary artery disease
CI	Confidence intervals
DS	Dual-sequence
DS-AIF	Dual-sequence-derived arterial input function
IQR	Interquartile range
LoA	Limit of agreement
MBF	Myocardial blood flow
MPR	Myocardial perfusion reserve
MRI	Magnetic resonance imaging
